# 1011. Case Study. Bovine Collagen Dermal Matrix Facilitates Re-epithelialization in Mixed Partial-thickness Burn Wounds: A Case Series

**DOI:** 10.1093/jbcr/irag033.199

**Published:** 2026-04-06

**Authors:** Hana Lopez-Quinones, Parsa Lotfi

**Affiliations:** Jacobi Medical Center, Bronx, NY; Jacobi Medical Center, Bronx, NY

## Abstract

**Patient Presentation (age range, injury details, relevant history):**

This case series describes two male patients aged 61 and 75 years, with multiple comorbidities and mixed partial-thickness thermal burns measuring approximately 100 cm^2^ and 300 cm^2^ on the left forearm and right lower extremity, respectively.

**Clinical Challenges:**

In burn and wound management, cutting-edge interventional studies and emerging technologies are most often applied to full-thickness and deep partial thickness injuries requiring surgical closure. In contrast, superficial and mixed partial-thickness wounds are typically managed more conservatively and receive less attention in interventional literature. This relative gap in evidence-based innovation may affect therapeutic options and delay healing for patients who fall into such category. By exploring new technologies such as bovine collagen based dermal matrix (BCDM), there is an opportunity to expand treatment paradigms for these patients. To illustrate the potential role of this approach, we present these two patients with mixed partial-thickness burns successfully treated with a novel BCDM.

**Management Approach:**

After initial wound care and debridement using a combination of sharp excision and hydrotherapy, a bovine collagen–based dermal matrix (BCDM) was applied at bedside directly to the wounds. Post-application wound management consisted of a primary non-adherent dressing layer, followed by compressive layers (Patient 1) or negative pressure wound therapy (NPWT) (Patient 2). Dressings for both were removed at 7 days post-application and were subsequently switched to antimicrobial ointment, non-adherent contact layer and compressive outer dressings. Both patients were monitored weekly for re-epithelialization, complications, and overall wound healing.

**Outcomes:**

Patient 1 achieved complete re-epithelialization within 4 weeks without complications or the need for autografting. Patient 2 was ready for autograft at first dressing change, however he was lost to follow up until he returned at 4 weeks with a fully healed wound. Post-application wound care was straightforward, and both patients reported satisfaction with cosmetic and functional outcomes, particularly with the avoidance of operative intervention.

**Lessons Learned:**

This case series demonstrates that BCDM can be an effective treatment option for mixed partial thickness burns, promoting timely healing while minimizing patient morbidity and possibly eliminating the need for grafting. These early findings suggest that collagen-based dermal matrices may expand the role of advanced biologics beyond full-thickness injuries, offering a valuable alternative in the management of selected partial-thickness burns.

**Applicability to Practice:**

The use of BCDM in outpatient or bedside settings may reduce the need for operative procedure and improve patient-centered outcomes in partial thickness burn management.

